# Hippocampal development and the dissociation of cognitive-spatial mapping from motor performance

**DOI:** 10.12688/f1000research.6966.2

**Published:** 2015-09-21

**Authors:** Bryan D. Devan, Christopher Magalis, Robert J. McDonald

**Affiliations:** 1 Psychology Department, Laboratory of Comparative Neuropsychology, Towson University, Towson, Maryland, 21252, USA; 2 Department of Neuroscience, Canadian Center for Behavioural Neuroscience, University of Lethbridge, Lethbridge, Alberta, TIK 3M4, Canada

**Keywords:** Spatial navigation, Place response, Hippocampal development, Multiple memory systems, Latent learning, Learning/performance distinction, Individual differences, Strategy selection

## Abstract

The publication of a recent article in *F1000Research* has led to discussion of, and correspondence on a broader issue that has a long history in the fields of neuroscience and psychology.  Namely, is it possible to separate the cognitive components of performance, in this case spatial behavior, from the motoric demands of a task?  Early psychological experiments attempted such a dissociation by studying a form of spatial maze learning where initially rats were allowed to explore a complex maze, termed “latent learning,” before reinforcement was introduced.  Those rats afforded the latent learning experience solved the task faster than those that were not, implying that cognitive map learning during exploration aided in the performance of the task once a motivational component was introduced.  This form of latent learning was interpreted as successfully demonstrating that an exploratory cognitive map component was acquired irrespective of performing a learned spatial response under deprivation/motivational conditions.  The neural substrate for cognitive learning was hypothesized to depend on place cells within the hippocampus.  Subsequent behavioral studies attempted to directly eliminate the motor component of spatial learning by allowing rats to passively view the distal environment before performing any motor response using a task that is widely considered to be hippocampal-dependent.  Latent learning in the water maze, using a passive placement procedure has met with mixed results.  One constraint on viewing cues before performing a learned swimming response to a hidden goal has been the act of dynamically viewing distal cues while moving through a part of the environment where an optimal learned spatial escape response would be observed.  We briefly review these past findings obtained with adult animals to the recent efforts of establishing a “behavioral topology” separating cognitive-spatial learning from tasks differing in motoric demands in an attempt to define when cognitive-spatial behavior emerges during development.

## Summary of new target findings

The study by Comba *et al.* ^1^ suggests a critical period of prenatal development (PND) in rodents during which neuronal mossy fiber growth in the hippocampus is associated with the emergence of spatial behavior. The researchers emphasize the PND 15–18 period where this growth connecting the dentate gyrus to the CA3 cellular field is most prominent. It is also noted in the introduction that such growth may be related to the finding that neurogenesis-based processes specific to the hippocampus are also associated with the emergence of spatial learning. The researchers describe a “behavioral topology” control in their research design to dissociate effects based on non-cognitive motor demands from true cognitive information processing, which was supported by their data analyses of place learning in the Morris water maze versus spatial exploration in a dry land task (with swimming being more difficult than common ambulation). Despite the difference in motor demands, there was a common emergence of spatial behavior proficiency on each task at PND20. The researchers also found that enhanced mossy fiber projections, revealed by synaptophysin staining in the CA3 region, preceded the emergence of spatial behavior. Developmentally-dependent functional changes in cFOS positive cells were increased in all hippocampal subregions measured, while training-dependent changes were restricted to the CA3 and CA1 regions for groups trained in the water maze. The researchers conclude that mossy fiber connectivity along with enhanced function of the hippocampus precedes the emergence of spatial behavior at PND20, confirming their hypothesis of a sensitive period for hippocampal growth and the emergence of cognitive-spatial function.

## Review of the broader issue: “Behavioral topology”

This research is very important and exciting when considering past studies of place learning ability in adult rats and research attempts to dissociate motor performance from true cognitive processing. In previous water maze studies of latent learning ^2, 3^ using passive placement on the goal in the water maze to view distal cues to form a cognitive map of the environment, mixed results and individual differences seem to obscure matters ^4– 8^, leading to one interpretation that movement and cognitive mapping may necessarily occur simultaneously, a finding that has been replicated in humans using a virtual version of the water maze ^9^. Hence, for the water maze at least, movement through the environment seems to be an important constraint on highly proficient spatial learning and navigation. The use of a separate dry land task by Comba *et al.* with less motoric demands seems to be in agreement with the difference in motor demands between the original rodent version of the task ^10^ requiring swimming and the human virtual version ^9^ using minimal hand/finger movements to navigate. The role of dynamic movement during spatial tasks and the motoric demands have been topics of intense interest with the role of the hippocampus as a substrate for cognitive mapping ^11, 12^, path integration ^13– 17^, or the conductor of a symphony of dynamic movement and mapping ^18^ as part of a larger neural network of brain systems ^19^ have all been hotly debated theoretically over the years. The separation of cognitive and motor performance associated with developmentally-specific changes in hippocampal circuitry by Comba *et al.* is an exciting finding that may have important implications for a central role of the hippocampus in cognitive-spatial information processing as it emerges early in development.

Consequently, the “behavioral topology” issue in the Comba *et al.* study, along with other aspects of their research design, is of critical importance in assessing the emerging role of the hippocampus in cognitive-spatial behavior. The following matters should be considered by all interested in this fascinating area of research in general, and in the Comba *et al.* study in particular.

## Specific considerations

1) The level of spatial proficiency in escaping to a hidden platform for PND20 rats given only eight trials in the water maze is not comparable to the asymptotic level of escape latency performance (< 10 sec) observed in most water maze studies after considerably more extensive training. The 1 day water maze training paradigm is likely tapping into ventral hippocampal function in which the animals are just approaching the general location. After more training, dorsal hippocampus forms a more precise representation of the location. The authors should discuss this work ^20^ and an analysis of their data (dorsal versus ventral) would be of interest.
2) On a related matter, escape is not required, and exploration of an object at a novel “place” in the dry land task is very different from the typical water maze procedure, involving the presence of a local cue or familiar beacon (with different motivation). Some might argue that the lack of “true” spatial proficiency in the water maze is a flaw or weakness of the study; however, given the focus on the emergence of spatial behavior, and that well-learned escape responses are dependent on other brain regions ^21, 22^ that contribute/correlate with movement parameters ^19^, it seems reasonable to expect less performance-wise using the escape latency measure than what is typically observed in most studies of this type.
3) Consequently, spatial bias on a probe test might be considered as an alternative measure in future studies as it does not depend on a well-learned escape response that may be less hippocampal-dependent and more closely approximates the dwell time that is measured on the spatial exploration task.
4) The object/place task is interesting. Integration with ideas about direct versus indirect measures of memory and the role of the hippocampus in one versus the other, and how these ideas relate to their different measures on this task would be of interest ^23^. For example, Moses *et al.* ^23^ suggest that rearing represents behavior directed specifically toward new distal cues in contrast to locomotion related to but not necessarily directed at novel cues. Rats with hippocampal damage lack the increase in rearing suggesting it is a direct measure of memory, whereas intact locomotion not directed at distal cues represents an indirect measure. Also, hippocampal lesions impaired all measures in the Morris water task, further supporting the dissociation of direct and indirect measures of memory.
5) There are other obvious differences between the two tasks. Water maze for example is not disrupted by disorientation procedures but a dry-land version of spatial localization is disrupted by disorientation ^24^. This work suggests that the representations are different as well, not just the behavioral topology. This should be discussed.
6) The number of rats in each group for the water maze task is quite low (n = 5/group).
7) A statement on the standardization of immunohistochemical procedures would be reassuring for those not familiar with the specific techniques used. Also, the use of an unbiased stereology technique should be considered.
8) It appears that different behavioral procedures may have been conducted at different institutions. A statement on the time of day of testing and other procedural controls would provide reassurance that there are no threats to internal validity.
9) More information on the recording and quantification of exploratory behavior (e.g., video recording, tracking, and interrater reliability) would be helpful for assessment and replication.
10) It is interesting that the researchers note that PND18 rodents traveled a longer distance in the novel relocation task then the other groups (even though apparently PND20 rats exhibited more exploratory behavior). This finding may warrant further discussion to support the argument that both tasks assess potentially related cognitive functions.
11) Reassurance that no statistical assumptions were violated (e.g., sphericity). Tukey HSDs are specifically based on studentized q-related statistics but t-tests were reported. Though this may have been simply an alternative method (e.g. regression-based) to report the Tukey post-hoc results, possibly Bonferroni t-tests with separate mean square error denominators may be optimal for potential corrections to assumption violations.

## Neural substrates of cognition and spatial performance

Following the lead of prior studies of spatial memory and hippocampal function using the radial arm maze ^25^, Morris initially used the approach of transecting the fornix/fimbria to disrupt hippocampal function ^26^, but only observed modest impairments in the water maze. Only later studies showed that direct neurotoxin lesions of the hippocampus produced severe impairments ^27, 28^. Sutherland and Rodriguez ^22^ showed that only complete transaction of the fornix/fimbria abolished both acquisition and retention of navigation to a hidden platform and detailed the effects of lesions to structures receiving input from the hippocampus via the fornix/fimbria, including severe impairments of postoperative acquisition produced by bilateral damage to the medial nucleus accumbens or bilateral damage to the anterior thalamic area with little effect on retention of preoperatively acquired place navigation. Also, damage to the medial septum or mammillary complex produced modest impairments evident only in postoperative acquisition. These findings were among the first to detail network connections involved in place navigation in the water maze.

A subsequent study ^21^ comparing preoperative fornix/fimbria versus caudate-putamen lesions revealed somewhat surprising results: place navigation escape latency performance was severely impaired by caudate-putamen lesions and only mildly impaired by fornix/fimbria lesions, manifesting on the latter trials of acquisition when controls had reached asymptotic performance (approximately under 10 sec/trial block). A detailed analysis of the animals’ behavior revealed that fornix/fimbria-lesioned rats used compensatory strategies, circumnavigating at a more-or-less constant distance from the pool wall until swimming into the platform, often without slowing down and anticipating climbing onto the refuge (failing to disinhibit the forepaws from the natural swimming posture). The fornix rats also showed evidence of using an angled trajectory from all start points that often led directly to the platform from one of the four start locations. The combination of these non-spatial strategies led to spared performance early in acquisition, consistent with the variable results obtained in previous studies. Despite the relatively less severe impairment on escape trials, fornix rats failed to show the normal spatial bias for the location of the platform when it was removed from the pool for the standard probe test. In contrast, the severe impairment in escape latency observed during acquisition trials for caudate-lesioned rats, which was due to prolonged thigmotaxis, did not interfere with their showing a near normal spatial bias on the probe test. These findings suggest that fornix rats were impaired at knowing where the platform was formerly located, while caudate-lesioned rats were impaired at implementing procedural aspects of the task (related to thigmotaxis) but not in knowing where. This conclusion directly contradicts the interpretation that fornix rats can learn a place response (knowing where) but have difficulty getting there (a motor impairment) ^17^ and is supported by human virtual navigation studies ^19, 29^ detailing a network of interactive brain systems. In comparison to the Comba *et al.* study, these findings suggest that a single day of place training with the primary performance measure of escape latency is less hippocampal-dependent than a probe test measure of spatial bias. Hence, a more impressive demonstration of when hippocampal-related spatial-cognition emerges during development would involve the use a probe test of performance in the water maze, as noted above.

The issue of spared early water maze acquisition following hippocampal damage and severe impairment due to caudate lesions suggests that the initial trials/day(s) of water maze training may primarily involve procedural learning as animals get accustomed to navigating in water and learn about the task demands. This may be related to the greater sensitivity of probe test performance to hippocampal damage ^21^, which typically occurs after considerable training. Analysis of micro-behaviors, such as the characteristics of swimming, pausing and path patterns may be more informative than the overall latency to escape, another reason why probe test behavior has the potential to reveal the specifics of spatial learning/memory, especially in a non-aggregated form that considers the temporal aspects of navigational behavior ^30, 31^.

## Individual differences in spatial behavior as a function of other factors

Given the fact that fornix-lesioned rats may use compensatory strategies during escape acquisition ^21^, and that a network of brain structures may contribute to spatial behavior ^19, 22, 29^, it is possible that individual differences may emerge during development. Individual differences in adult rats during place navigation and following a latent learning test in a novel environment suggested differential influence of the stimulus control of spatial behavior, with entrance into a room representing a polarizing cue for some individuals ^4^. Such changes may occur later in development and involve cholinergic markers in other brain structures such as the striatum, with stable measurements observed in the hippocampus ^32^.

A form of individual difference, sex/gender, is another area where mixed results have been observed in the water maze ^33^ and have been shown to depend on strain and volumetric brain differences, including the hippocampus and its subregions, prefrontal cortex areas and the amygdala ^34^. Behaviorally, the largest sex differences have been reported during the initial trajectory phase of a trial ^35^, which may depend on effective processing of distal features of the environment in planning appropriate navigational behavior. In another study young male and female rats were equally proficient in finding the platform during training trials, however probe tests showed that young male rats had better knowledge of the platform's precise location and was correlated with larger basal forebrain cholinergic neurons compared to females ^36^. Further, there was no sex difference in aged rats that exhibited an overall spatial learning impairment, however aged males now had smaller cholinergic neurons whereas no change was observed in females. These results reveal a complex interaction between sex, age and spatial behavior. Comparing performance on different spatial tasks in humans, including virtual water maze, Astur *et al.* ^37^ concluded that even after equating factors such as motivation, stress and motor demands, procedural demands of the tasks may nevertheless lead to differential strategy selection during spatial memory, and suggested that researchers use caution when utilizing different tasks interchangeably as tests of spatial memory. Hence, assuming that differences in the motoric demands of two tasks isolates spatial cognition may not account for a myriad of other differences that could potentially influence performance.

## Conclusion

Although the main contribution of the Comba *et al.* study is in the potential advancement of our knowledge on hippocampal plasticity related to the emergence of cognitive-spatial behavior during a developmentally-specific sensitive period, the importance of integration across networks and neural systems should not be lost in the reductionism. For example, recent findings show that water maze performance may change the functional connectivity between subregions of hippocampus and striatum in female rats and humans ^38, 39^. Perhaps future work may focus on a comparison of sensitive periods across these systems to define functional connectivity among cellular networks of brain systems. Focus may also be expanded to different time points in the life cycle, to not only the developmental emergence of cognitive function but also the stabilization, maintenance and eventual decline, having potential translational value in the treatment of neurodegenerative disease.
