# Supplementary material for: Hippocampal development and the dissociation of cognitive-spatial mapping from motor performance
Source: F1000Res. 2015 Sep 21;4:625. Originally published 2015 Aug 25. [Version 2] doi: 10.12688/f1000research.6966.2 (PMC4648208; doi:10.12688/f1000research.6966.2)
Supplement: Peer review history

# Peer review: Referee response for version 1

**DOI:** 10.5256/f1000research.7502.r10249

**Published:** 2015-09-10

**Authors:** Jeffrey Long

**Affiliations:** 1 Laboratory of Behavioral Neuroscience, Neurocognitive Aging Section, National Institute on Aging, Baltimore, MD, USA

This Correspondence article was inspired by the author’s recent review of an F1000Research report examining the temporal relationship between hippocampal anatomical development and spatial cognition. The first part of this opinion article summarizes their previous review and the second part nicely expands into a more theoretical discussion of the relationship between the cognitive and motor demands of spatial memory tasks. The manuscript calls attention to several important factors to be considered when critically evaluating performance in spatial memory tasks and I recommend approval.

I have read this submission. I believe that I have an appropriate level of expertise to confirm that it is of an acceptable scientific standard.

# Peer review: Referee response for version 1

**DOI:** 10.5256/f1000research.7502.r10254

**Published:** 2015-09-10

**Authors:** Derek Hamilton

**Affiliations:** 1 Department of Neurosciences, University of New Mexico, Albuquerque, NM, USA

This brief correspondence was motivated by a recent F1000Research report by Comba *et al. *in which the development of mossy fiber projections and associated functional alterations in the CA3 and CA1 subfields were argued to precede the emergence of spatial cognition as measured at PND16, 18, or 20 in two tasks that differ in the motor and motivational properties. Based on the observations that improved latencies in the Morris water task and detection of changes in object location were observed only at PND20 the authors of the original target article conclude that the aforementioned neurobiological development precedes emergence of the purely spatial cognitive abilities in question (without respect to specific motor skills). Devan *et al. *utilize this conclusion as a point of departure for discussion of a long-standing issue in the study of spatial navigation and its development concerning the relative contributions of cognitive and motor skills. This is perhaps best represented in the literature on “latent learning” in the water task as well as the broader literature on neural systems involved in motor and spatial learning to which the authors have made important contributions. The authors briefly provide a summary overview of the target article findings, a review of the broader issue of “behavioral topology”, an enumerated list of specific considerations (many related specifically to the target article), a brief review of some key neurobiological findings relevant to the central theme of the manuscript, and a final section on the significance of individual differences in performance. I am inclined to recommend approval because this is a brief correspondence piece that nicely frames what has been an important, if not central, thematic issue in the field of spatial behavior for many years in the context of the recent report by Costa *et al*. This is a reasonable opinion/correspondence article that emerged from a prior published F1000Research review which may influence subsequent thinking and research on the ontogeny of spatial navigation and its neural bases and does not include specific technical/methodological aspects that require review. I do, however, offer several recommendations for improving the manuscript that the authors could consider.

1. Perhaps the most significant issue is that the basic premise is based on the conclusion that spatial proficiency emerges in tasks that vary in motor and motivational demands around PND20. As is noted, this conclusion is based on latency data in the water task that suggest improvement in performance at PND20 not observed at younger ages. The path length data, however, suggest that the animals are swimming roughly 4 times the diameter of the pool in order to escape, regardless of age. Thus, the suboptimal reductions in latency among the PND20 rats do not reflect direct trajectories to the escape platform characteristic of “optimal” spatial learning, which complicates interpretation. This is likely related to the limited number of training trials that were used, which the authors address. Given the importance of this conclusion, additional reference to prior work that used more training trials (e.g., 12-24) concentrated on a single day may provide better support for a developmental emergence of spatial learning around PND19-21.  
2. The authors draw attention to several important aspects of the Costa *et al. *article that are worthy of consideration and frame these in the context of general points for which readers interested in the topic should be aware. This is an important component of the manuscript and, overall, these points are well reasoned and accurate. In the spirit of highlighting points of importance for studies of this type I would suggest the authors consider a few additional points (or modifications) that, in my opinion, are important for developmental studies of navigation in rats. These include : 1) considering development of thermoregulation abilities (e.g., Brown and Whishaw, 2000; Akers and Hamilton, 2007) which could potentially affect performance (in both motor and cognitive domains) differently in dry and wet tasks, 2) the apparatus size (e.g., Carman and Mactutus, 2001), and 3) to expand on point 3 from the authors’ enumerated list, the use of tasks/measures that are sensitive to the detection of constituent cognitive processes during development (see e.g., Akers *et al.*, 2011, as data from that study suggest that distal visual cues can control some aspects of spatial behavior in the water task as early as PND17, but definitely not on PND16).
3. Regarding point 4 in the authors’ list, I agree that this is interesting, however, some brief elaboration on this point could help frame this suggestion for readers unfamiliar with the direct/indirect distinction as described in Moses *et al*. 
4. Under “Neural substrates of spatial cognition”, the work described in the paragraph beginning “A subsequent study …” should include a citation for clarification.
5. Perhaps the most important single statement in this correspondence is in the conclusion that attention to the development of functional interactions among the broader circuitry involved in spatial navigation not be lost in the context of conclusions about specific behavioral, cognitive, or neurobiological elements. The potential influence of this suggestion for subsequent research and thinking on this important issue could be strengthened by emphasizing this point earlier in the manuscript.

 

Some minor issues

 

- In the first sentence of the “Summary of New Target Findings” the word “month” should be omitted
-  “Typology” or “topology” : The authors use both “behavioral typology” and “behavioral topology” in the manuscript.
- In the sentence (page 4, col 2 of pdf) beginning “Analysis of micro …”, “then” should be “than”

I have read this submission. I believe that I have an appropriate level of expertise to confirm that it is of an acceptable scientific standard.
